# Supplementary material for: Templated paired helical filament formation and tau fold-dependent inclusion morphologies in primary neurons
Source: bioRxiv. 2026 Oct 5:2026.09.30.755038. Preprint. [Version 1] doi: 10.64898/2026.09.30.755038 (PMC13691994; doi:10.64898/2026.09.30.755038)
Supplement: Supplementary file 1 [file media-1.pdf]

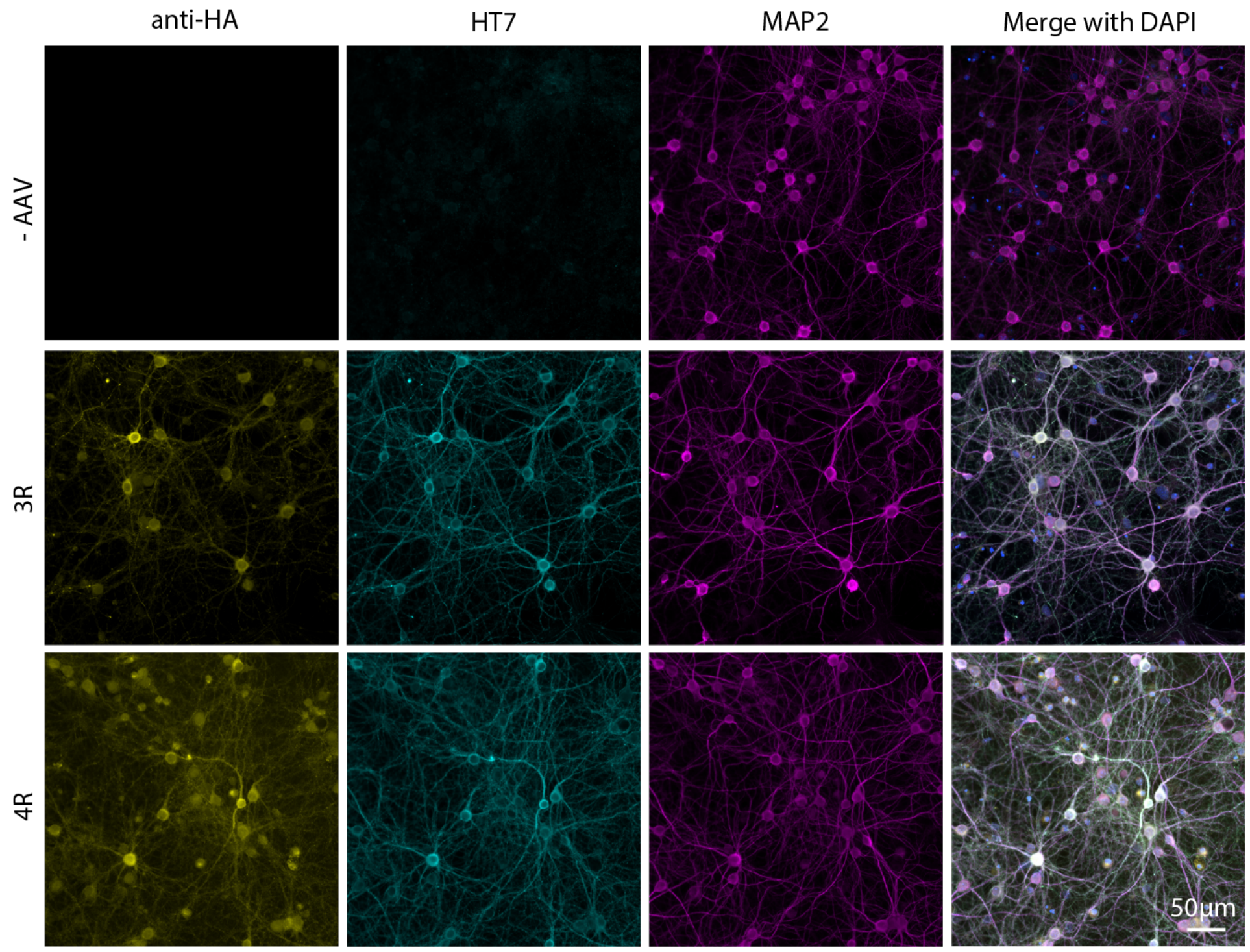

Figure S1: Expression of human tau isoforms in primary mouse cortical cultures following AAV transduction.

Immunofluorescence microscopy of primary cultures at DIV14 transduced with AAV1/2-hSyn-HA-0N3R and AAV1/2- hSyn-HA-0N4R, fixed with 4% paraformaldehyde, using anti-HA, anti-tau (HT7), and anti-MAP2 antibodies.

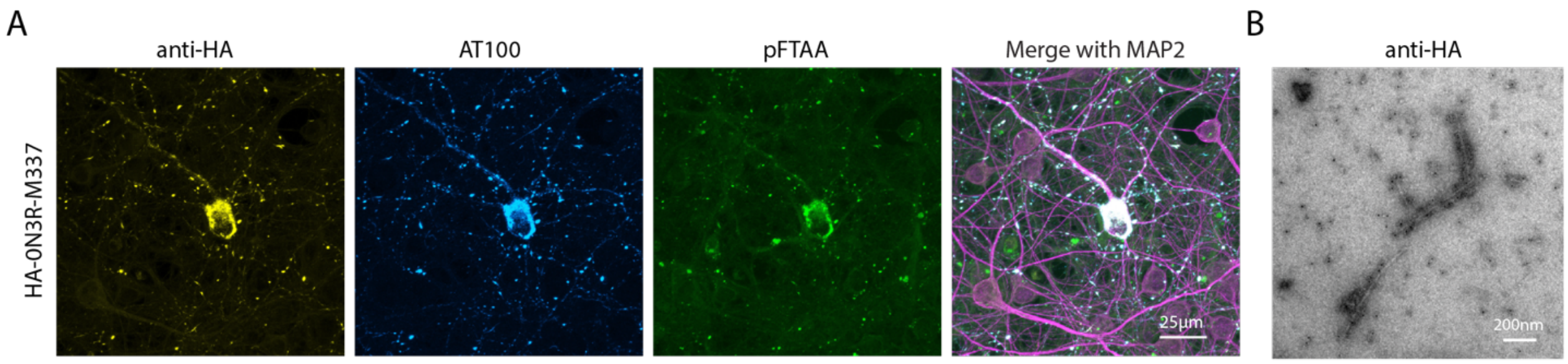

Figure S2: AD seeded filamentous inclusions form in neurons expressing HA-0N3R-M337 tau.

A) Representative fluorescence images of primary cultures at DIV21 expressing HA-0N3R-M337 tau and treated with AD seeds. B) Negative stain immunoelectron micrographs of sarkosyl-insoluble extracts using anti-HA antibodies showing gold-labelled filaments.

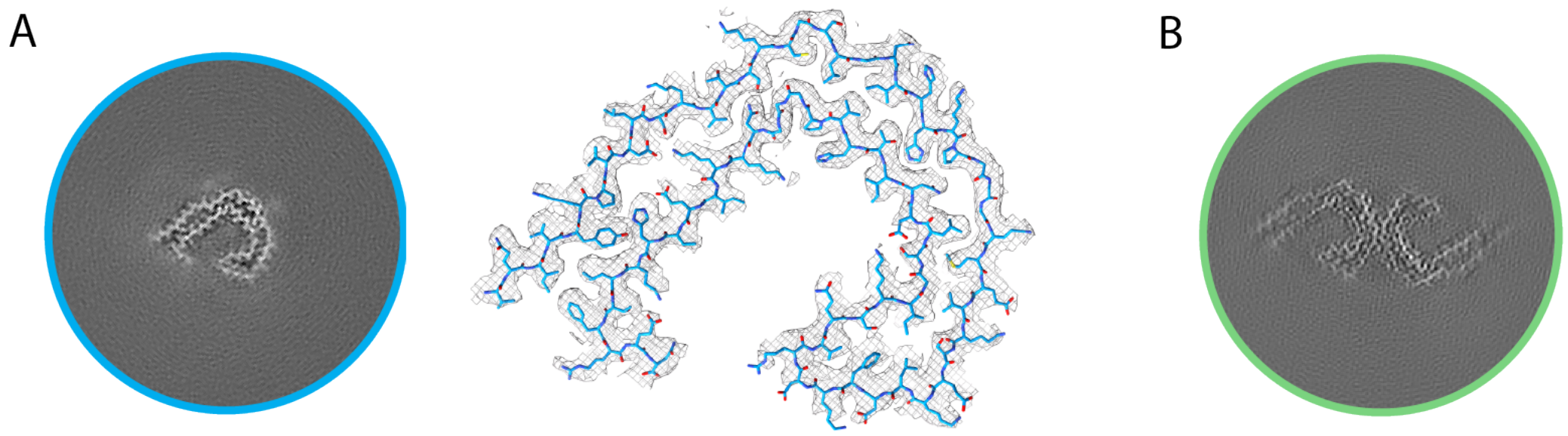

Figure S3: Cryo-EM density map of single protofilament with the Alzheimer tau fold and cryo-EM density map of PHF from the AD seeds.

A) Cross-section of reconstructed density of singlet filament extracted from primary cultures expressing HA-0N3R-M337 tau and treated with AD seeds (0.2mg/cm^2). Singlet protofilaments with the Alzheimer tau fold represented 18% of filaments and were solved to a resolution of 2.9 Å. B) Cross-section of reconstructed density of tau filaments extracted from the frontal cortex of AD case 2. The PHF structure was solved to a resolution of 2.7 Å.

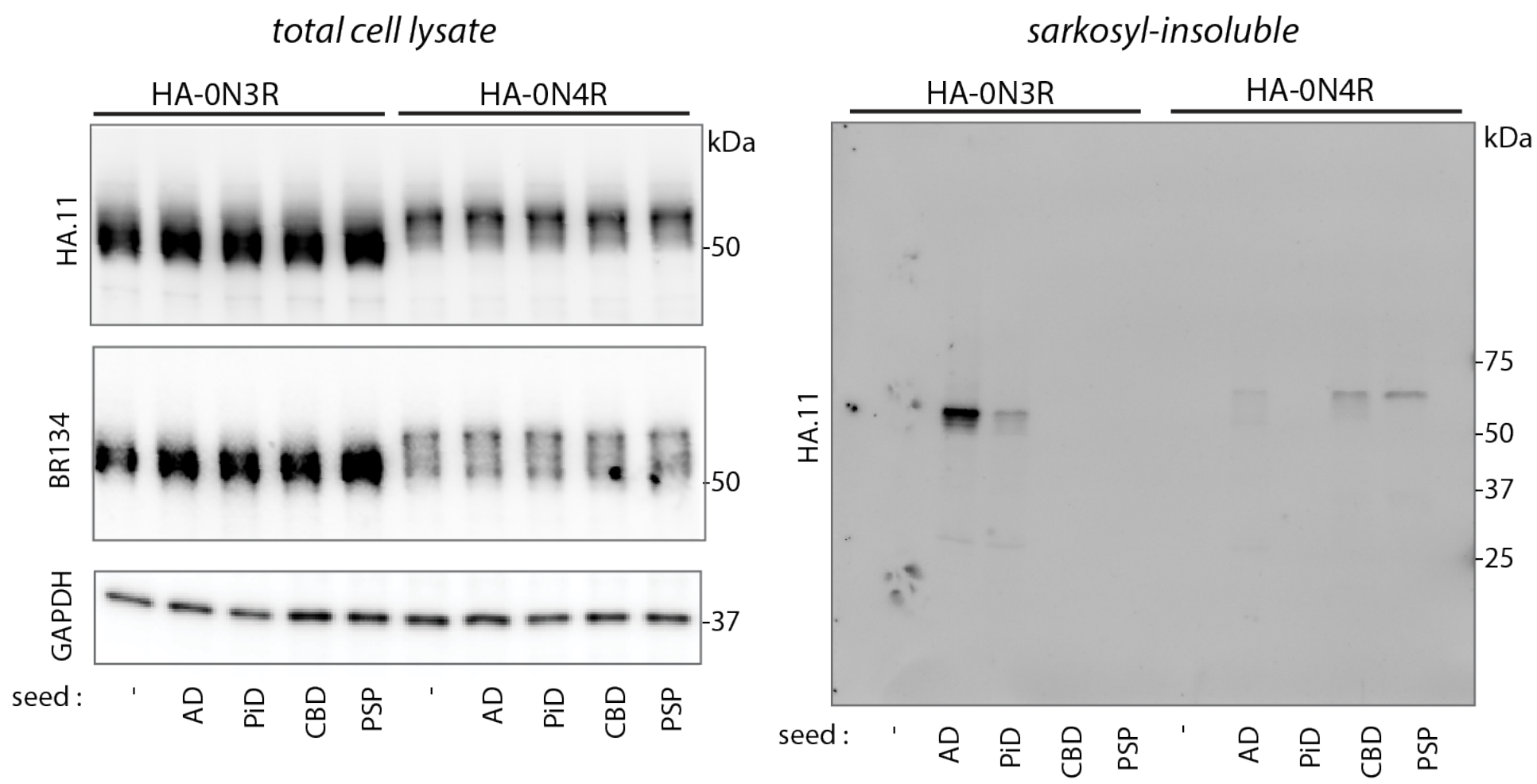

Figure S4: Seeded aggregation in primary neurons recapitulates disease-specific tau isoform selectivity.

Immunoblot analysis of total cell lysates and sarkosyl-insoluble fractions of neurons expressing HA-0N3R or HA-0N4R tau and treated with AD, PID, CBD or PSP seeds.

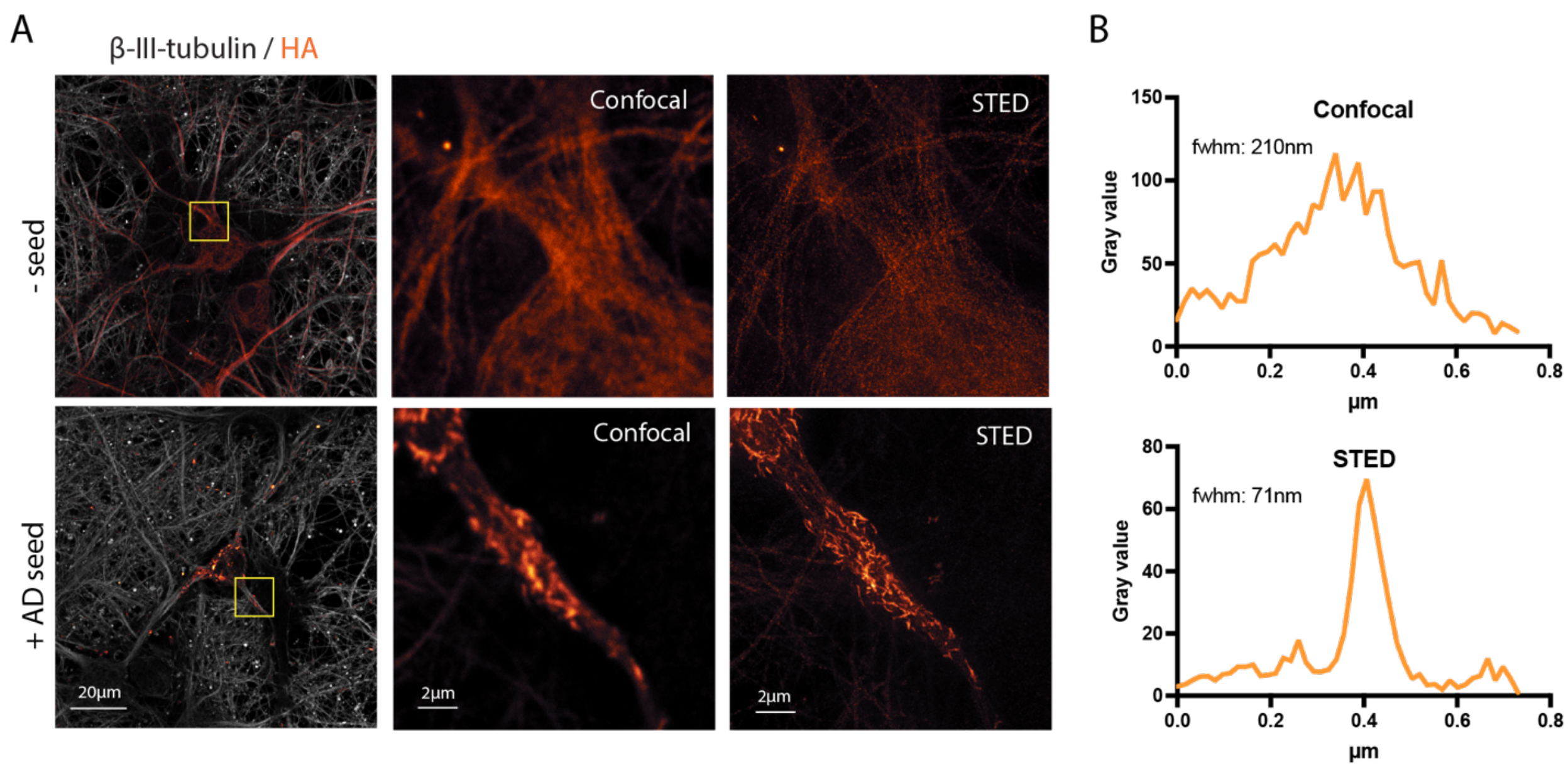

Figure S5: Super-resolution microscopy imaging of seeded tau inclusions.

A) Representative confocal and STED images of primary neurons treated +/- AD tau seeds at DIV14 and labelled with β-III-tubulin and anti-HA. B) Representative cross-sectional profile of a filamentous tau inclusion measured by confocal microscopy (top) or STED (bottom).

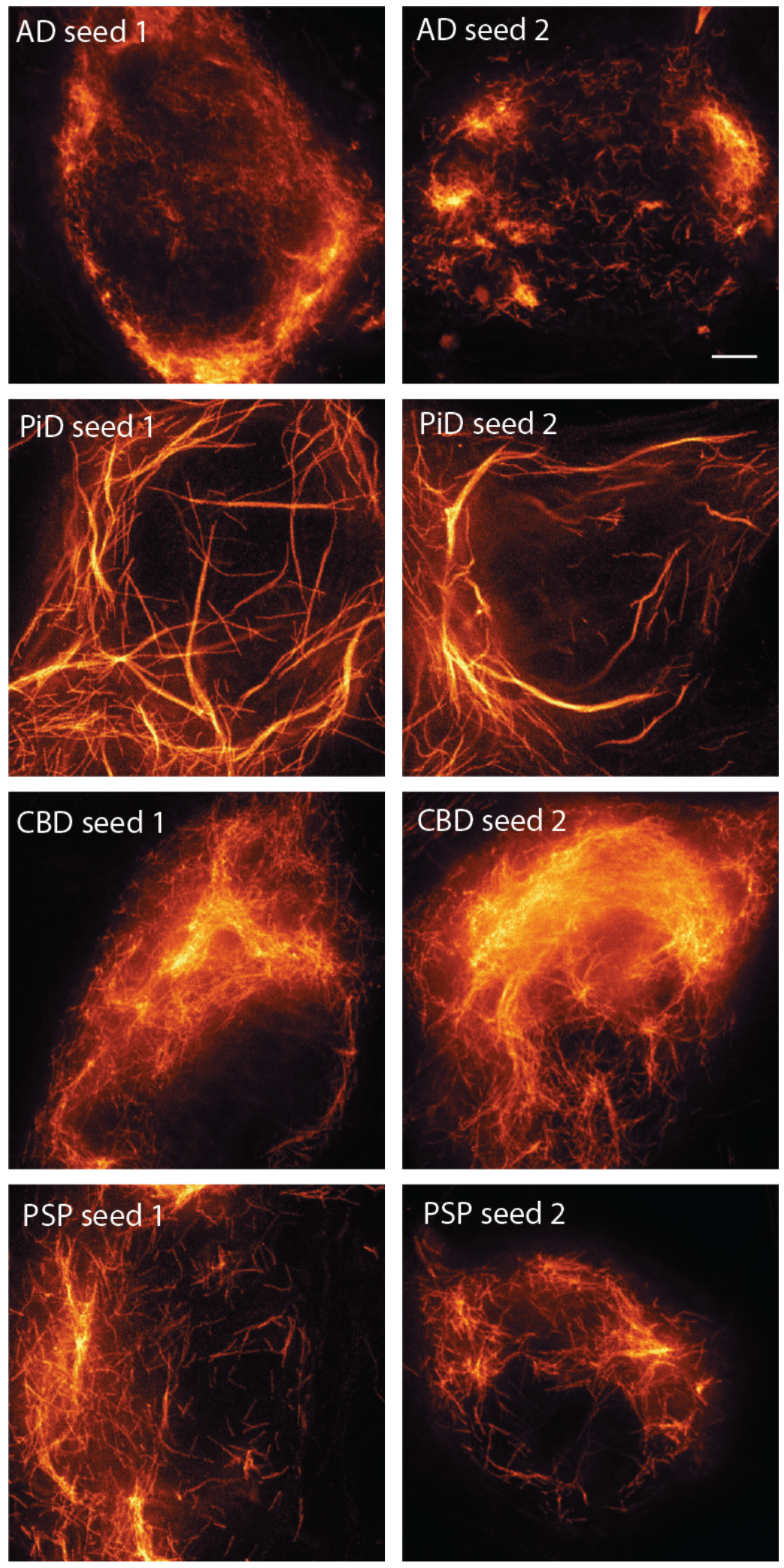

Figure S6: Tauopathy-derived seeds produce distinct intraneuronal inclusion morphologies.

STED images at DIV21 of seeded neuronal tau inclusions following the addition of seeds from two cases of AD, PiD, CBD, or PSP. Scale bar, 2μm

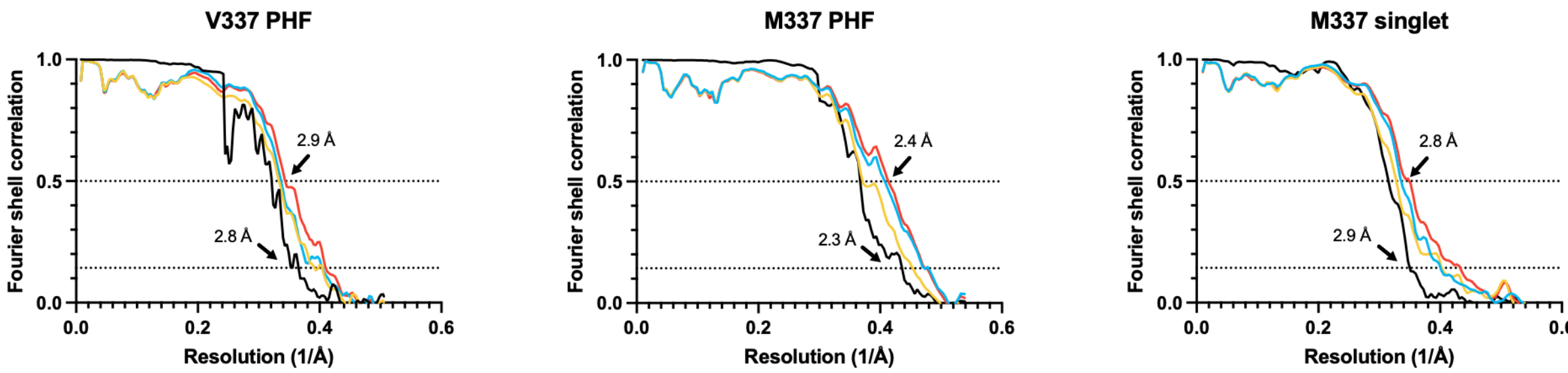

Figure S7: Resolution estimates for the M337 PHF, M337 singlet, and V337 PHF. Solvent-corrected Fourier-shell correlation (FSC) curves between independently refined half-maps are shown in black; FSC curves between the refined model and the reconstruction from all particles are shown in red; FSC curves between a model refined against half map 1 against half map 1 are shown in blue; FSC curves of the same model against half map 2 are shown in yellow. Resolution estimates are indicated at thresholds of 0.143 for the half-map FSCs and 0.5 for the model-to-map FSCs

Table S1. Cryo-EM data acquisition and structure determination

	V-PHF	M-PHF	M-Singlet
Data collection			
Microscope	Titan Krios		
Voltage (kV)	300		
Detector	Falcon 4i		
Energy filter slit width (eV)	-	10	
Magnification	96,000	130,000	
Electron exposure (e-/Å^2)	40		
Defocus range (μm)	-1.0 to -2.5	-1.0 to -2.0	
Pixel size (Å)	0.824*	0.926	
Data processing			
Symmetry imposed	C1		
Initial particle images (no)	973,577	985,972	985,972
Final Particle image (no)	271,595	176,278	125,248
Map resolution (Å) (FSC threshold 0.143)	2.8	2.3	2.9
Helical rise (Å)	2.41	2.43	4.88
Helical twist (°)	179.46	179.28	-1.353
Refinement			
Initial model	6HRE	ModelAngelo	
Model resolution (Å) FSC threshold	2.9	2.4	2.8
Map sharpening *B* factor (Å^2)	-56.8	-53.8	-51.3
Model composition			
Non-hydrogen atoms	3528	3522	1761
Protein residues	462	456	228
B factors (Å^2)			
Protein	76.7	86.7	85.5
R.m.s. deviations			
Bond lengths (Å)	0.0097	0.0101	0.0111
Bond angles (°)	1.80	1.75	2.19
Validation			
MolProbity score	1.68	1.36	1.84
Clashscore	3.10	1.51	5.71
Poor rotamers (%)	0	0	0
Ramachandran plot			
Favored (%)	92.00	92.57	90.54
Allowed (%)	8.00	7.43	9.46
Disallowed (%)	0	0	0

* The V-PHF map was refined at a downscaled pixel size of 0.988 Å.

Movie S1: Long-term live-cell imaging of progressive tau inclusion formation in primary neurons. Wide-field images of seeded neurons expressing dendra2-0N3R and seeded at DIV03, taken every 6 h in an Incucyte S3 Live-Cell system.

Movie S2. High-resolution live-cell imaging of tau inclusions in primary neurons. Confocal imaging (maximum intensity projections) taken of AD seeded neurons expressing dendra2-0N3R at DIV10 in one field of view over 12 h. Inset: a neuronal cell body with multiple independent events of fluorescent tau inclusion formation.
